# Perception of Tobacco Counseling and Cessation among Dental Practitioners

**DOI:** 10.1155/2021/6692525

**Published:** 2021-03-16

**Authors:** Jazia A. Alblowi

**Affiliations:** Department of Periodontology, Faculty of Dentistry, King Abdulaziz University, Jeddah, Saudi Arabia

## Abstract

**Objective:**

To investigate the knowledge and practice of tobacco cessation and counseling (TCC) among dental practitioners and their attitude and perceived barriers.

**Methods:**

A cross-sectional study targeted licensed dental practitioners in Jeddah, Saudi Arabia. Participants answered a pretested and validated self-administered questionnaire consisted of demographic data; smoking status; knowledge of tobacco hazards, attitude, and practice; and perceived barriers of tobacco cessation counseling.

**Result:**

Among the total sample of 529, response rate was 72.2% (mean age (34.20 ± 9.38 years), males (42.4%), and current smokers (23.8%)). Only 13.2% received formal training on TCC. Around (57.1%) reported smoking of dental team as an obstacle for TCC. Half of the participants (49.9%) reported patient's resistance as barrier to TCC. Others (45%–48%) reported insufficient time, knowledge, or training for TCC. Professional responsibility and willingness to provide cessation services constituted the highest median.

**Conclusion:**

The majority showed willingness to participate in TCC. Lack of training, smoking status of providers, females, inadequate materials, and patients' resistance were the most common barriers. Education and training on TCC are recommended and should be allowed as a routine practice in dentistry.

## 1. Introduction

Tobacco is known for its negative effect on general and oral health [1]. It is associated with oral precancerous lesions, oral cancers, periodontal diseases, and tooth loss [2]. Smoked and smokeless tobaccos not only negatively affect oral health but also affect clinical management of oral diseases and conditions [2]. However, it is one of the most modifiable risk factors of disease worldwide [3, 4]. Many studies have shown that former smokers have better oral health than current smokers [2, 5, 6]. Strong recommendations were made for the clinicians to suggest smoking cessation to smokers to decrease the negative effect on periodontal health as well as to prevent the risk of oral cancers [2, 7].

Tobacco cessation reduces the prevalence and progression and improves treatment of periodontal disease [8, 9]. There is evidence that supports substantial significant benefits of tobacco cessation on oral health at all ages [2].

Tobacco counseling has been a point of concern since around 1970s when smoking cessation guidelines were suggested to dentists by an air force dental clinic in order to help patients quit smoking [10]. Tobacco cessation counseling (TCC) was defined as a technique for patient's counseling by health professionals to guide them quit tobacco habit [11].

Two nationwide cross-sectional studies conducted in Saudi Arabia in 2018 reported high prevalence of smoking (32.5% among males and 3.9% among females) [12].

A report from WHO on global tobacco epidemic 2019 showed that compliance in Saudi Arabia in terms of smoke-free policies was moderate and taxation constituted 68.1% [13], while evidence from a study conducted in the United Kingdom suggested that behavioral intervention by oral health practitioners may be effective in tobacco cessation [14]. Although from a different country, but the experience is worth to be applied to the health profession worldwide.

The specific situation of dental professionals places them in good position to offer preventive care [15, 16]. Furthermore, the multiple visits needed for dental treatment provide dentists with great opportunities to communicate with patients and reinforce the concept of tobacco cessation [17].

The World Health Organization (WHO) recommended that dentists and other oral health professionals should be involved in helping patients to quit smoking, and that tobacco cessation should be part of the practice of dentistry [1].

Because little is known about the TCC application in dental practice by dental practitioners in Jeddah, Saudi Arabia (SA), the aim of this study was to investigate the knowledge, attitude, and practice of tobacco cessation and counseling among dental practitioners and to assess the perceived barriers and limitations to its application.

## 2. Methods

A cross-sectional study design was approved by the Research Ethics Committee, Faculty of Dentistry, King Abdulaziz University (KAU) (protocol number: 033-2015). The questionnaire was developed after thorough literature review and was reviewed by three experts in tobacco cessation practice to ensure that the measured variables adequately cover the measured content (content validity). A pretest study was conducted on eighty dental practitioners after revision of the questionnaire to check the clarity and feasibility of the survey and to test for face validity. The participants in the pilot study were asked to provide their feedback, and modifications were done accordingly. A total sample size of 411 dentists were previously determined based on the following: total number of dental GPs, specialist working in Makkah Region (which involves dentists from both Makkah and Jeddah Cities without splitting of data), and faculty members of KAU (*N* = 1295) as calculated from a document extracted from data of the Saudi Commission for Health Specialties [18], yielding 4% margin of error for the attitude score among dentists [19] and a confidence level of 95% . The sample size was calculated using the online website https://Raosoft.com [19, 20].

A purposive sample including both the Faculty of Dentistry of King Abdulaziz University (KAU) members(19.7%) and the practicing dentists from outpatient clinics (19.4%) and from hospitals with the largest numbers of dentists (60.9%) was collected to provide a reasonable balance between all dental health settings. The study targeted dentists licensed by the Saudi Commission for Health Specialties from all districts of Jeddah City; from each district, at least two dental treating centers were visited and all dentists available were approached. The final study was conducted by distributing the structured questionnaire on 529 licensed dental practitioners who worked as full or part time in Jeddah, Saudi Arabia, and did not share in the pilot study.

All participants received the questionnaire with a covering letter in which the rationale for the study was clarified and verbal informed consent was obtained from each participant.

The final questionnaire consisted of 6 parts: demographic data, detailed smoking status, knowledge of tobacco effect on general and oral health, attitude and professional responsibility, practice of and perceived barriers to tobacco cessation, and counseling in the dental practice. Internal consistency reliability of knowledge questions was 0.825, 0.634 for general harmful effects, 0.730 for dental harmful effects, 0.510 for tobacco use in KSA, and 0.838 for treatment modalities. Cronbach's alpha value for overall attitude was 0.882 with reliabilities of 0.840 for professional responsibility towards smoking cessation, 0.810 for extent to which TCC falls within the scope of dental practice, and 0.736 for willingness to provide cessation services. Reliability values for beliefs and practices were 0.736 and 0.858, respectively.

Statistical analyses were performed using the Statistical Package for the Social Sciences (SPSS) version 22 (IBM Corp., Armonk, NY, USA). Descriptive data were represented in percentages and stratified with the different relevant variables, and median values were calculated for dentists' knowledge, attitude, and practices. Logistic regression analysis was performed for the effect of independent variables related to TCC.

## 3. Results

Among the total number approached (529), only 382 responded (72.2% response rate) with no significant difference in response rate by position or gender. This was detected by reviewing the incomplete questionnaires and comparing them to the participants' complete formats. The mean age was 34.20 age range, working hours ± 9.38 years, and males constituted 42.4% (Table 1). General practitioners were 43.1%, while 20.1% had master's degree and about 36.3% had PhD degrees. The average working hours is 8.73 + 2.15 working hours and 8.27 + 5.91 patients seen/day. Current smokers constituted 23.8%, mainly cigarettes and shisha, while 20% reported family smoking. Only 13.2% received formal training on TCC.

As a justification of noninclusion of TCC in dental practice, the highest percentage of dentists (57.1%) said that some dental team members may be a smoker themselves (Table 2). Almost half of the sample participants perceived patient's resistance as a great barrier to TCC. Other considerable percentages (45%–48%) reported lack of time or necessary material for TCC and that patients would not be happy talking about smoking in dental practice. Almost one-third reported lack of sufficient knowledge (Table 3).

**Figure 1:**
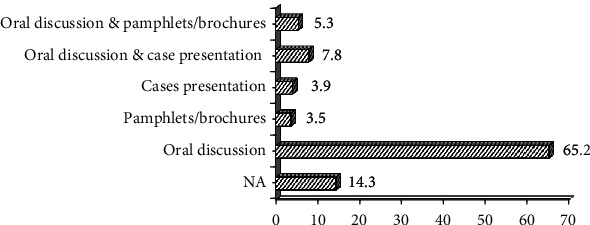
Dentists' materials used in their practice to aid in TCC.

The commonly used tool for TCC as stated by dentists (64.2%) is oral discussion, and a small percentage used case presentation (3.9%) and/or pamphlets (3.5%) (Figure 1).

**Figure 2:**
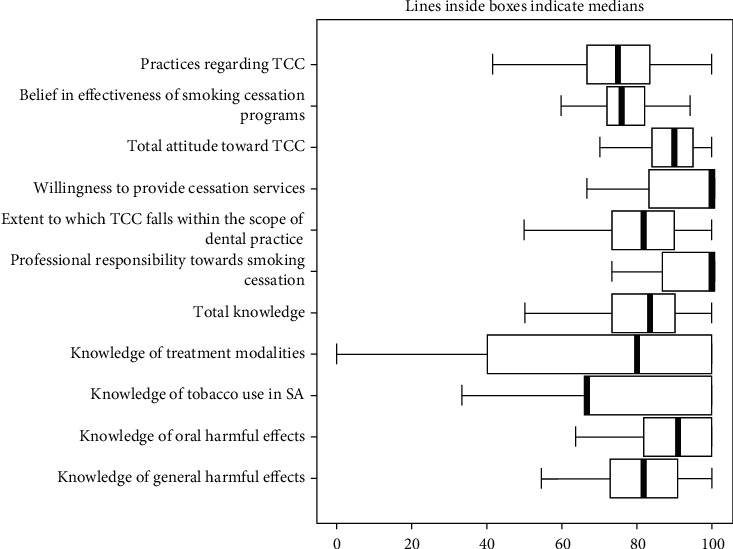
Knowledge, attitude, belief, and practices related to TCC of dentists.

Medians and distribution of dentists' knowledge, attitude, belief, or practice related to TCC are illustrated by the box plots in Figure 2. The highest medians were for professional responsibility towards smoking cessation and willingness to provide cessation services (almost 100% of maximum score). The next highest median score was that of knowledge of oral harmful effects and total attitude toward TCC (around 90%). The lowest median scores were observed with knowledge on tobacco smoking in SA and practices of TCC (66.7% and 75%, respectively).

**Figure 3:**
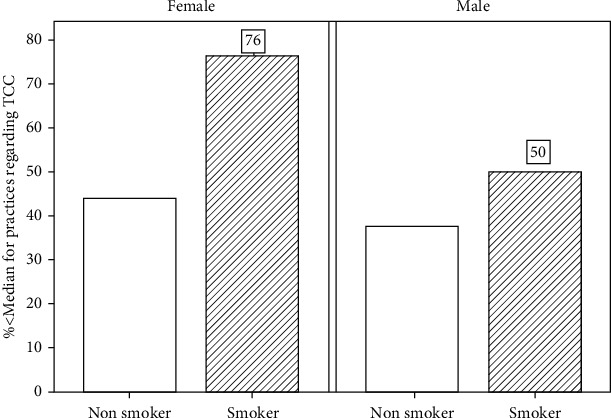
Practices of TCC by gender and smoking status.

Factors significantly related to lower scores (less than median) on knowledge, attitude, belief, or practice are presented in Table 3. General practitioners (GPs) and males were less likely to have negative attitude regarding TCC, and those with negative beliefs are more likely to score lower on attitude (adjusted OR = 7.77 (95% CI: 4.58-13.19). Beliefs related to TCC are significantly associated with number of patients seen/day, knowledge, and attitude, whereas lower belief scores were observed with a smaller number of patients seen/day and lower knowledge and attitude scores. TCC practice scores were lower among current smokers, with lower knowledge or attitude scores. Smoking ∗ gender interaction was found to be significant in the model for practices indicating that the relation with smoking is modified with gender.  Figure 3 shows that though the percentage of poor practices (<median) was higher among smokers than nonsmokers, the gap between them is wider among females more than males. The percentage of poor practices among female smokers is higher (76%) than the corresponding percentage among male smokers (50%).

## 4. Discussion

Different studies have suggested the need for involvement of different oral health professionals in tobacco cessation programs [11, 21]. Tobacco use and cessation counseling among dental or oral health practitioners was investigated in different communities [16, 17, 21–27]. In Jeddah, to the best of our knowledge, this is the first study which investigated tobacco cessation and counseling among dental practitioners on different positions.

The percentage of current smokers among participating dentists in the present study was 23.8%, mainly cigarettes and shisha with 20% reported family smoking. This is smaller than what was reported in previous studies [22, 23].

Regarding the barriers to participate in TCC, the highest frequency (57.1%) of dentists considered that some members of the dental team may be smokers as a barrier. The results of the study have already shown that 23.8% were current smokers which gives them a good reason for their perception, or maybe they expressed their own status. However, overcoming it by constructive discussion with the dentists themselves on the importance of their participation in TCC should be considered. This factor was also mentioned by another researcher [22].

Lack of time was the second cited barrier for nonparticipating in TCC (48.4%) which was agreed upon in the previous study [22]. This factor could be critical for dentists working in private clinics; however, for the public sector, TCC should become an integral part of the dentist's schedule and duties and be given sufficient time to perform it [22]. Lack of information was reported by almost one-third of the practitioners. These findings are supported by in other studies where inadequate time, lack of reimbursement, and inadequate training in counseling were the major barriers reported for TCC practice [22, 27, 28]. However, authors reported that time should not be considered as a barrier as three minutes is sufficient for counseling and brief intervention from health professionals can lead to 10-15% quit rate [28, 29]. Some countries realized the importance of this matter and have called to encourage dentists to be involved in TCC [30, 31].

With regard to the participants who reported nonparticipation fearing that the patient will not be happy with the procedure (22.8%), a Saudi study conflicted this belief and reported that patients documented that participation in TCC by dentists is a feature for quality dental services [22].

As oral discussion was the most highly reported by 65.2% of the participants, and considered as a very useful method for one-to-one education and convincing, however, it can be augmented by many other aids such as posters, pamphlets, or case presentations which were reported by a very small percentage of the participants (Figure 1). Therefore, these aids should be made available and dentists be trained on utilizing them to overcome resistance to tobacco cessation.

Almost all dental practitioners reported professional responsibility towards smoking cessation and willingness to provide cessation services which is similar to other reports where dentists believed that significant support should come from dentists [23] and dentists considered counseling very important [32]. The participating dentists also showed a high median score of knowledge on the harmful effects of tobacco which may have contributed to their willingness to provide cessation counseling. This attitude should be considered very positive provided that facilities be available and barriers be overcome for the dentists to provide maximum cooperation in TCC programs.

The lowest median value for knowledge of participating dentists was related to the questions on tobacco use in Saudi Arabia. This may reflect a shortage of their time to acquire information on important health problems of their own local community, but draws attention to conduct more in-service education programs for professionals. Meanwhile, the internal consistency value calculated for this group of questions was low (Cronbach's alpha was 0.51) which may point at misunderstanding of the respondents and to interpret the responses with caution. Subsequently, this can be considered a limitation of the study results.

Lower belief scores also were related to a fewer number of patients seen per day, although it is opposite to expectation but could be related to less interaction with patients' problems and habits.

When relating smoking status to willingness to provide tobacco cessation counseling, there was highly significant association reported previously in some studies [22], but not others [24, 26]. Also, less positive attitude towards smoking cessation counseling was reported by some smokers [25]. This may be related to a feeling of disappointment of the smoker dentists due to their own inability to stop the habit themselves or a deceiving personal experience, whereas in the meantime, they are required to provide advice to their patients.

Smoking status of the practitioners was linked to the poor practice (less than median). Moreover, percentage of poor practices among female smokers was higher than the corresponding percentage among male smokers. This may be because a Saudi female still has some concerns about admitting smoking. Consequently, a smoking Saudi female dentist would be embarrassed to provide advice on a habit that she herself practices while knowing it not only presents a health hazard but also is rejected by the community.

In the present study, current smokers, with lower knowledge or attitude scores, had lower TCC practice scores. Similarly, smoker dentists showed less willingness to engage in tobacco cessation activities. This was supported by previous research [22] and is explained by the logical relation between a person's knowledge and attitude affecting his or her practices.

The importance of having this study carried out is reassured by the campaign sponsored by the Centers for Diseases Control and Prevention (CDC) in an attempt to decrease heart attack and strokes in the coming five years, and dental practice offers a great opportunity for patients' education and practice [18].

Furthermore, optimum patient care should be a goal for all health professionals, which necessitates that tobacco cessation counseling should be carried out by them all [18].

## 5. Conclusion

According to the results of the current study and with consideration to the limitations of a cross-sectional questionnaire-based survey that addressed a critical health habit, it was shown that almost all of the dental practitioner showed professional responsibilities and willingness to participate in tobacco counseling. Lack of knowledge, smoking status of the providers especially females, inadequate materials, limited training, and patients' resistance were reported as the most common barriers. A structured and timed protocol of the TCC should be developed and refined, to integrate this concept in educational programs. Undergraduate training and in-service continuing education and practice should be delivered to all dental practitioners, and sufficient time should be allowed for them for its routine application.

## Figures and Tables

**Table 1:** General characteristics of dentists of the study sample.

Variables		Total no. of dentists (*n* = 382) 100%
`		34.20 (9.38)
Age categories (years, %)	<30	41.5%
	30-39	28.9%
	40-49	21.2%
	50-60	8.4%
Gender (%)	Male	42.4%
	Female	57.6%
Position (%)	GP	43.1%
	Specialist	56.9%
Degree (%)	BDS	43.7%
	Master	20.1%
	PhD	36.3%
Setting (%)	Outpatient	19.4%
	Hospital	60.9%
	Academic	19.7%
Current smoking (%)	Yes	23.8%
Working hours/day (mean (SD))		8.73 (2.15)
Number of patients seen/day (mean (SD))		8.27 (5.91)
Formal training on TCC (%)	Yes	13.2%

**Table 2:** Perceived barriers to tobacco smoking cessation counseling.

Perceived barriers to TCC		%
Extent to which patient resistance might be a barrier to TCC in the dental setting	Great	49.9
	Moderate	43.1
	Low	7.0

Reasons for not including TCC in dentists' practice	Do not have enough knowledge	32.5
	It is not the dental practitioner's job to do this	12.1
	All patients know the negative effect of smoking	12.5
	Only patients with systemic problems need TCC	2.9
	Patients will not respond positively	16.5
	Patients will not be happy talking about smoking in dental practice	22.8
	Lack of time	48.4
	Lack of necessary materials	45.3
	Some dental team member may be a smoker themselves	57.1

**Table 3:** Logistic regression analysis of factors associated with dentists' knowledge, attitude, belief, and practices related to TCC.

	Knowledge		Attitude		Belief		Practice	
	OR	95% CI	OR	95% CI	OR	95% CI	OR	95% CI
GP			0.32^∗^	0.19-0.54				
Male			0.49^∗^	0.27-0.89				
Current smoker							4.60^∗^	1.80-11.78
Gender smoking^∗^							0.29^∗^	0.09-0.96
No. of patients seen/day					0.89^∗^	0.42-0.96		
Knowledge (<median)					2.65^∗^	1.59-4.40	1.72^∗^	1.07-2.79
Attitude (<median)					7.69^∗^	4.53-13.05	3.70^∗^	2.22-6.15
Belief (<median)			7.77^∗^	4.58-13.19				

^∗^ *p* < 0.05 (significant).

## Data Availability

The data used in this study is available upon request from the author.
